# STM tip-assisted engineering of molecular nanostructures: PTCDA islands on Ge(001):H surfaces

**DOI:** 10.3762/bjnano.4.104

**Published:** 2013-12-18

**Authors:** Amir A Ahmad Zebari, Marek Kolmer, Jakub S Prauzner-Bechcicki

**Affiliations:** 1 Research Centre for Nanometer-scale Science and Advanced Materials (NANOSAM), Institute of Physics, Jagiellonian University, Reymonta 4, 30-059 Krakow, Poland

**Keywords:** molecular nanostructures, scanning tunneling microscopy, tip-induced processes

## Abstract

Islands composed of perylene-3,4,9,10-tetracarboxylic dianhydride (PTCDA) molecules are grown on a hydrogen passivated Ge(001):H surface. The islands are studied with room temperature scanning tunneling microscopy and spectroscopy. The spontaneous and tip-induced formation of the top-most layer of the island is presented. Assistance of the scanning probe seems to be one of the factors that facilitate and speed the process of formation of the top-most layer.

## Introduction

On-surface engineering of molecular nanostructures is one of the key elements for many forthcoming technologies. A wide range of possibilities is explored to search for an efficient, precise and cheap strategy for the fabrication of various organic nanostructures. Recently, there has been an increasing interest in the field of molecular self-assembly-based processes as a means of organic nanostructure formation [1–3]. As such, self-assembly allows for obtaining nanowires, two dimensional lattices, molecular islands, and molecular mono- and multilayers with a high yield. The resulting structures are often stable and almost perfect. The implementation of bottom-up self-assembly-based methods in an industrial process may require, however, the reshaping and tailoring of the structure with precise top-down methods to obtain the desired shape and properties. Scanning tip induced processes may serve as such a step to adjust the final form of the molecular nanostructure.

The design and formation of a molecular device is a key element of its successful operation. However, the desired properties of the device may be severely hampered by its environment, e.g., dangling bonds of a semiconducting substrate surface or electrical contact with a metallic substrate. There have been developed several strategies to minimize or even eliminate the influence of the underlying substrate on a molecular nanostructure on-top of it [4]. From an industrial perspective, a very promising approach is to cover the chosen substrate by an additional ultra-thin buffer layer, i.e., either a few monolayers of an insulator (e.g., NaCl on metal surfaces [5–13] or KBr on InSb [14–15]) or even a single layer of an atomic or molecular species (e.g., passivation of Si or Ge surfaces [16–19]). Such an extremely thin interlayer not only electronically decouples on-top adsorbed molecular species, but additionally may dramatically enhance the mobility of the molecules and increase their chances to self-assemble and form molecular nanocrystals [9–11,20]. For the purpose of the present study it is very convenient to focus on the hydrogen passivation of Si and Ge surfaces. It has been shown in case of Si(001) [17], Si(111) [18] and Ge(001) [19] surfaces that such a passivating layer electronically decouples the molecule from the substrate and increases their mobility.

In this article, high-resolution scanning tunneling microscope (STM) measurements of self-assembled perylene-3,4,9,10-tetracarboxylic dianhydride (PTCDA) molecular islands on a hydrogen passivated germanium surface, Ge(001):H, are presented. The application of bias voltage pulses in STM allows for the modification of the islands. We found that the presence of a scanning tip of the tunneling microscope facilitates and speeds the formation of a new full top-layer of the island.

## Results and Discussion

**Figure 1:**
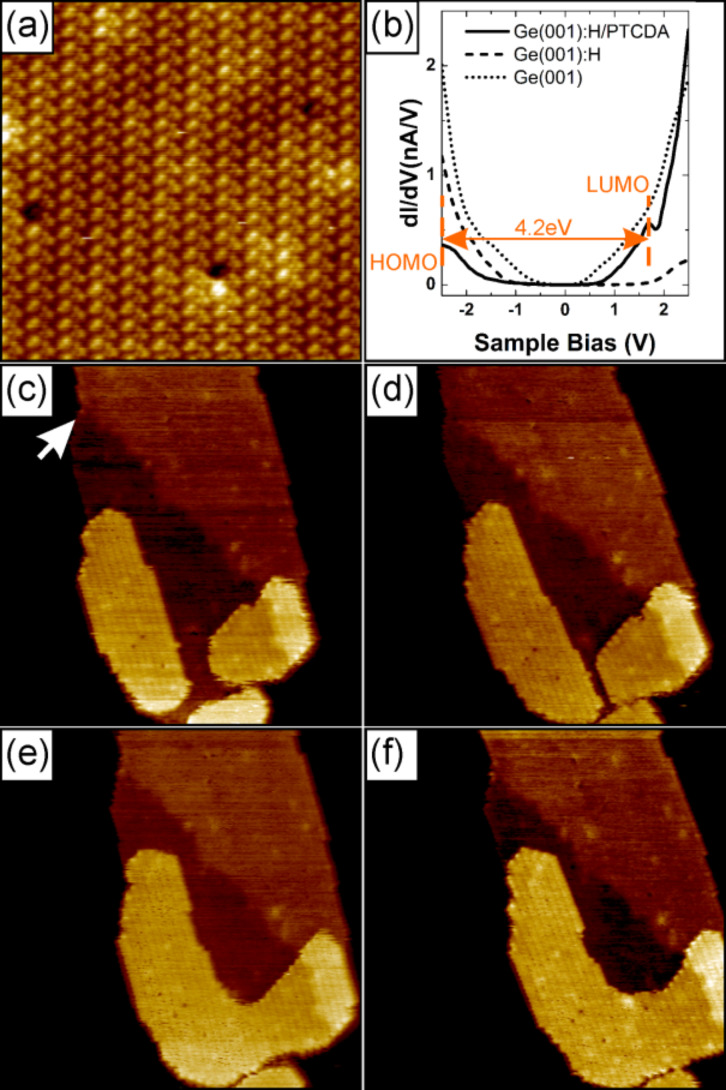
(a) High resolution STM image on top of a PTCDA island, 25 nm × 25 nm, showing the herringbone structure. (b) STS curves for Ge(001), Ge(001):H and PTCDA molecular island. (c)–(f) Four successive scans of the same area to illustrate the gradual growth of top layer, scan size 100 nm × 100 nm. White arrow marks the change in contrast on the island attributed to the underlying step-edge of the Ge(001):H substrate. For each STM image ((a) and (c)–(f)) the scanning parameters are *I* = 10 pA, *U* = +2 V.

Due to the presence of a passivating hydrogen layer on the Ge(001) surface, molecule–substrate interactions are significantly weakened and molecules are extremely mobile. The imaging of a single molecule at room temperature is impossible. At the high coverage, however, the accumulation of the PTCDA molecules is dominated by molecule–molecule interactions and molecular islands are formed. The islands grow in the Volmer–Weber mode. The density of the islands is 2.5 × 10^9^ cm^−2^ for coverage of 0.7 ML. Approximately 60% of the islands exhibit a strip-like hexagonal shape with two long edges and four short ones. It is noteworthy that molecular islands quite often extend in one direction over 100 nm and more, traversing several substrate terraces without any influence to their structure. It is possible to achieve high-resolution images on top of the islands in rt STM (see Figure 1a). These images show that the islands have crystalline character, and the top-most layer closely resembles the herringbone structure found for the (102) plane of PTCDA bulk crystal [21–22]. Similar arrangements have been reported for the Si(001):H/PTCDA system [20]. Most of the islands have a height of 2.1 nm, what corresponds to 6 molecular layers.

Insight into the electronic structure of the studied system is obtained by rt STS measurements (see Figure 1b). For a bare germanium surface a band gap of ≈0.2 eV is obtained, in fair agreement with literature data [23–25]. A hydrogen passivated surface exhibit a band gap of ≈0.85 eV, similarly to a recently reported value obtained from low temperature measurements [25]. The energy gap between the highest occupied molecular orbital (HOMO) and the lowest unoccupied molecular orbital (LUMO) of a PTCDA island on Ge(001):H is measured as 4.2 eV. The latter value corresponds well with results reported for thick films (>5 nm) [26–29]. The electronic properties of the PTCDA islands are very different from the underlying passivated germanium, and there are no other features in the bias window from −2.5 V to 1.7 V (corresponding to the semiconducting energy gap of PTCDA molecules) of the STS curves. This means that the electronic structure of PTCDA is unperturbed by the electronic properties of the underlying substrate.

Figure 1c–f show a set of four consecutive scans of the same area on top of the PTCDA island. The change of the contrast in the middle of each of the scans (see white arrow in Figure 1c) originates from the step-edge of the underlying Ge(001):H surface. It may serve as a reference point for the observed evolution of the top-most layer. Every scan took 9 minutes (taking one image from the top to the bottom includes forward and backward scans). In the first scan from the set (Figure 1c) one can see that the starting structure of the top-most layer of the molecular island was composed of separate features, each of which has a height of one monolayer. On a subsequent scan (Figure 1d) one can observe a gradual growth of these features, eventually leading to their coalescence into one object (Figure 1e) that continues to gradually grow (Figure 1f). Typically, the morphology of PTCDA islands are stable during a STM/STS characterization. We assume that the presented evolution of the top-most molecular layer was probably unintentionally induced during a “cleaning” procedure of the scanning probe, i.e., by application of high voltage pulses.

**Figure 2:**
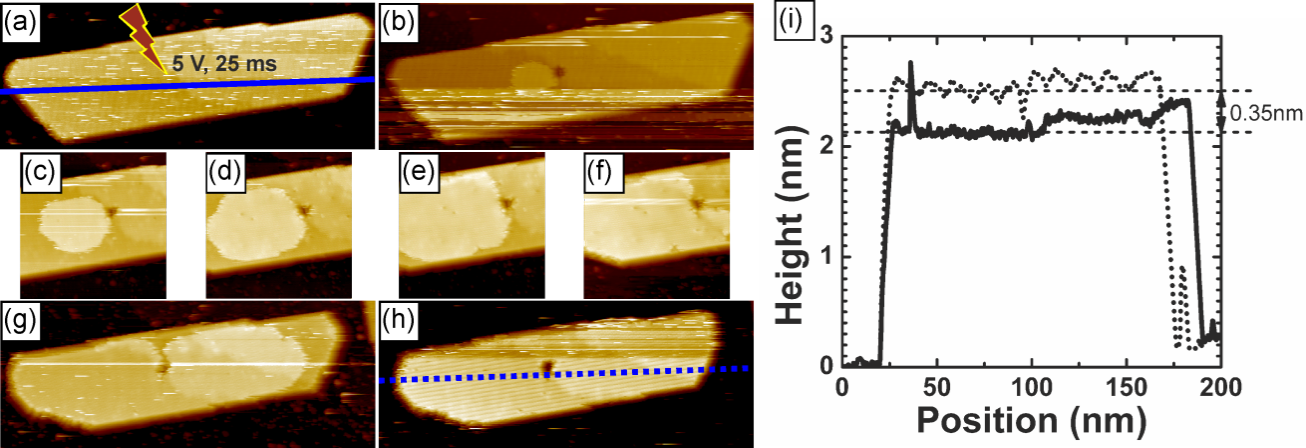
(a)–(h) A sequence of scans showing tip-initiated growth of the top-most layer of the PTCDA molecular island. Size of images:(a),(b),(g),(h) 70 nm × 180 nm, (c)–(f) 70 nm × 70 nm. Scanning parameters are *I* = 10 pA, *U* = +2 V. Blue solid and dotted lines mark positions at the cross-section height profiles. (i) The cross-section height profiles of the same island before and after the completion of the top-most layer indicated by solid and dotted lines (see (a) and (h) for position), respectively.

To investigate the initiation of growth of the top-most layer of an island by well-defined conditions we applied a bias voltage pulse of 5 V for 25 ms in the middle of the island (Figure 2a). As a consequence we observed a hole at the position of the pulse, and ad-molecules gathered around (Figure 2b). Consecutive scans (Figure 2c–h) show a gradual growth of the top-most layer. In the course of time a new full top layer is formed with the pulse-made hole remaining unhealed. The speed of the growth of the top-most layer is approximately 124 nm^2^/min.

The edges of the hole play the role of nucleation sites for the created layer. Energy barriers at the rim of the hole, i.e., Ehrlich–Schwoebel barriers, are too high to be crossed by diffusing molecules at rt, even in the presence of the field created by the scanning tip, and the molecules prefer to diffuse laterally within the same layer instead of moving downward to fill in the hole [30]. The height of the island changes by 0.35 nm, as can be inferred from a comparison of the cross-section profiles of the island before and after the top-most layer was formed (Figure 2i). The observed change in the height corresponds well to the distance between molecular planes in the [102] direction of the PTCDA bulk molecular crystal [20]. The edges of lower laying molecular sheets are a plausible source of molecules for the newly formed top-most layer. The edges observed on the scans are quite often fuzzy and change their shape during the manipulation (Figure 2). Moreover, after the adlayer formation the island considerably decreased its lateral dimension (Figure 2i).

**Figure 3:**
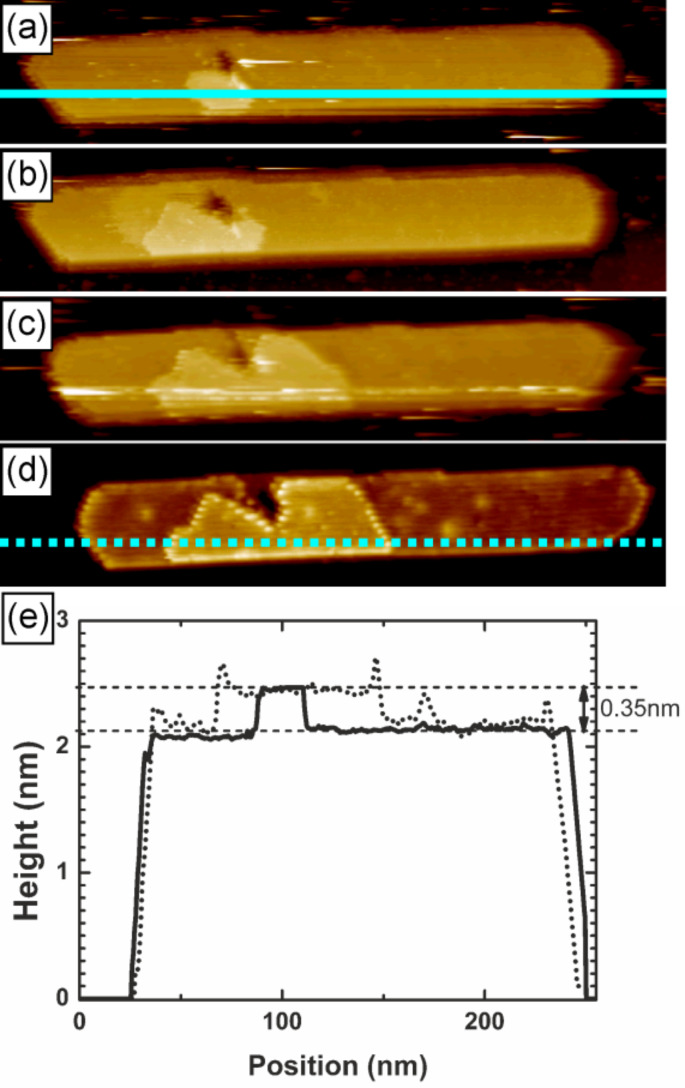
(a)–(d) A sequence of scans showing a partial growth of the top-most layer without continuous presence of the scanning tip. The light blue solid and dotted lines mark the positions of the cross-section height profiles. Scan size: 50 nm × 250 nm. Scanning parameters are *I* = 3 pA, *U* = +2 V. Scan (d) is taken 2 h later than scan (a). (e) The cross-section height profiles of the same island before and after the formation of the top-most layer indicated by solid and dotted lines (see (a) and (d) for positions), respectively.

In both discussed examples the island was continuously scanned during formation of the top-most layer. To shed some light on the role of the scanning tip in the process we performed a follow-up experiment. We applied a bias voltage pulse on top of an island (7 V, 25 ms). In Figure 3a we present the image of the island immediately after the pulse. Then, we retracted the tip for 10 minutes. After that time only a small increase in the size of the newly formed top-most layer was observed (Figure 3b). Thereafter, we retracted the tip again, this time for 20 minutes. Similarly, only a slight change in the size of the new adlayer was recorded (Figure 3c). We decided to retract the tip once more, for 30 minutes. And again, a minor change in the size was observed. In Figure 3d we present the image of the top-most layer of the island 130 minutes after the pulse. The total time elapsed from the pulse includes 60 min when tip was retracted and 70 min spent on scanning. From the inspection of the cross-section height profiles in Figure 3e it is clear that there was only a single-layer-height structure formed on the top of the island that did not exceed over the whole island. The overall average speed of growth of the observed structure in this case is approximately 13 nm^2^/min.

Most probably, the three dimensional mesa-like shape of the PTCDA crystalline nanoislands grown on Ge(001):H surface results from an efficient ascending interlayer transport. The configuration of molecules in a layer is determined, to some extent, by strain in the layer. Roughly speaking, the less strained a layer is the more relaxed molecules are in it. Yet, the amount of stress encountered by the molecules in the layer depends on the distance from the island–substrate interface. Thus, the further away from the interface the layer is, the less strain it experiences [31]. Consequently, binding energies on the edges of lower lying layers are smaller than binding energies on the edges of higher lying layers. Therefore, molecules attached to the edges of lower lying layers prefer to ascend and attach to more favorable sites on higher laying layers. Due to the applied bias voltage pulses we created new edges on the top-most layer offering convenient adsorption sites with high binding energies. Thus, we expect that an ascending interlayer transport is responsible for the newly grown top-most layer. The presence of the scanning tip seems to enhance that kind of process. Most probably, the presence of the electric field generated by a biased STM probe efficiently decreases the corresponding energy barriers for an ascending interlayer molecular transport. Hence, a continuous scanning of the island after pulsing allows for formation of the top-most layer roughly one order of magnitude faster than has been observed for intermittent scanning (compare results presented in Figure 2 and Figure 3). We would like to stress that this result is of qualitative character only, as many different parameters (e.g., tip composition/geometry, current set-point, bias voltages, sample temperature, etc.) may play a role in setting the final growth rate.

It is rather expected that in our experiment the scanning probe is coated with molecular material. One could then argue that the direct deposition from the tip should also significantly contribute to the observed growth of the top-most layer. However, if such a mechanism was the main source of the material it would usually lead to unstable imaging conditions. On the contrary, we observe growth of the top-most layer without disturbances typically associated with scanning tip modifications. Additionally, the direct deposition from the tip would not necessarily result in changes in the lateral dimensions of the islands, which is seen in each of the analyzed events of the growth (see for example cross-section height profiles in Figures 2i and 3e).

## Conclusion

We presented a rt STM/STS study of PTCDA crystalline nanoislands on a Ge(001):H surface. The high-resolution measurements revealed that the top-most layer has a structure closely resembling the herringbone structure found for the (102) plane of PTCDA bulk crystal. Spectroscopic data showed no influence of the substrate on the electronic properties of the islands. The crystalline nanostructures can be easily modified by the scanning probe, and the presence of the tip seems to be one of the factors that facilitate and speed formation of the top-most layer of the island. This feature may be a suitable supplementary step for self-assembly-based methods to fine-tune the final form of the molecular nanostructures of interest.

## Experimental

The experiments were carried out in a multi-chamber ultra-high vacuum system equipped with variable temperature STM (Omicron GmbH). The base pressure in the system was in the low 10^−10^ mbar range, with the exception of the microscope chamber where the pressure was 4–5 × 10^−11^ mbar. Atomically flat Ge(001) surfaces were prepared by a few cycles of simultaneous annealing of the samples at 780 °C (as measured by infrared pyrometer) and ion beam bombardment (1 keV Ar^+^, at 45° off-normal) for 20 minutes. The ion current density was approximately 0.3 μA/cm^2^. The samples were held to slowly cool down to room temperature at a rate of 0.1 A/min. To obtain a passivated surface the Ge(001) samples were exposed to hydrogen atoms provided by a homebuilt hydrogen cracker. The partial hydrogen pressure in the chamber was kept at 4–5 × 10^−7^ mbar for 2.5 hours, and the sample was kept at 200 °C. The PTCDA molecules were deposited with the use of a standard effusion cell (Kentax GmbH) at 310 °C on the sample, which was kept at room temperature. The molecular flux was controlled by a quartz-microbalance. STM measurements were carried out in constant current mode at room temperature (rt) by means of electrochemically etched tungsten tips as probes. Scanning tunneling spectroscopy (STS) measurements were carried out at rt. The STS data were averaged over 2500 curves taken from a grid covering a 10 × 10 nm^2^ surface area. The differential tunneling conductance (d*I*/d*V*) as a function of the sample bias *V* was obtained numerically from the *I*–*V* curves.
